# Assessment of Microhardness of Conventional and Bulk-Fill Resin Composites Using Different Light-Curing Intensity

**DOI:** 10.3390/polym15102250

**Published:** 2023-05-10

**Authors:** Selma Jakupović, Nedim Pervan, Elmedin Mešić, Alma Gavranović-Glamoč, Anita Bajsman, Enis Muratović, Lejla Kazazić, Alma Kantardžić-Kovačević

**Affiliations:** 1 Department of Restorative Dentistry and Endodontics, Faculty of Dentistry with Clinics, University of Sarajevo, 71000 Sarajevo, Bosnia and Herzegovina; jakupovic_selma@yahoo.com; 2 Department of Mechanical Design, Faculty of Mechanical Engineering, University of Sarajevo, 71000 Sarajevo, Bosnia and Herzegovina; mesic@mef.unsa.ba (E.M.); muratovic@mef.unsa.ba (E.M.); 3 Department of Prosthodontics, Faculty of Dentistry with Clinics, University of Sarajevo, 71000 Sarajevo, Bosnia and Herzegovina; alma.glamoc@gmail.com (A.G.-G.); lejla.kazazic@gmail.com (L.K.); 4 Department of Dental Morphology with Dental Anthropology and Forensics, Faculty of Dentistry with Clinics, University of Sarajevo, 71000 Sarajevo, Bosnia and Herzegovina; anita.bajsman@gmail.com; 5 Faculty of Dentistry with Clinics, University of Sarajevo, 71000 Sarajevo, Bosnia and Herzegovina

**Keywords:** resin composites, bulk-fill composites, microhardness, high-intensity curing, filler content

## Abstract

(1) Background: This study evaluates the effect of a conventional/low-voltage light-curing protocol (LV protocol) (10 s with 1340 mW/cm^2^) and high-voltage light-curing protocol (HV protocol) (3 s with 3440 mW/cm^2^) on the microhardness (MH) of dental resin-based composites (RBCs). Five resin composites were tested: conventional Evetric (EVT), Tetric Prime (TP), Tetric Evo Flow (TEF), bulk-fill Tetric Power Fill (PFL), and Tetric Power Flow (PFW). (2) Materials and Methods: Two tested composites (PFW and PFL) were designed for high-intensity light curing. The samples were made in the laboratory in specially designed cylindrical molds; diameter = 6 mm and height = 2 or 4 mm, depending on the type of composite. Initial MH was measured on the top and bottom surfaces of composite specimens 24 h after light curing using a digital microhardness tester (QNESS 60 M EVO, ATM Qness GmbH, Mammelzen, Germany). The correlation between the filler content (wt%, vol%) and the MH of the RBCs was tested. For the calculation of depth-dependent curing effectiveness, the bottom/top ratio for initial MH was used. (3) Conclusions: MH of RBCs is more dependent on material composition than on light-curing protocol. Filler wt% has a greater influence on MH values compared to filler vol%. The bottom/top ratio showed values over 80% for bulk composites, while for conventional sculptable composites, borderline or suboptimal values were measured for both curing protocols.

## 1. Introduction

Composites are defined as materials that are formed by two or more materials that have different physical and chemical properties and have superior properties compared to their parts [1,2]. Resin-based composites (RBCs) are widely used for the development of dental biomaterials. The main components of RBCs include organic matrix resin, inorganic part-fillers, coupling agents, and initiators of the polymerization process [3,4,5]. Resins are composed of a mixture of two or more monomers to achieve balanced functionalities in workable rheology and the desired mechanical properties before and after curing [6]. They mainly consist of the bifunctional monomers Bis-GMA and UDMA (urethane dimethacrylate), and due to their high viscosity, TEGDMA (triethylene glycol methacrylate) is used as a diluent. Bis-EMA (bisphenol A polyethethylene glycol dimethacrylate) is added to improve handling properties and reduce polymerization shrinkage [3].

The addition of various inorganic filler contents, such as silica glass, quartz, ceramic, metal, and pre-polymerized particles, in diverse shapes and sizes, can enhance the mechanical properties [7,8,9,10] of the dental composites while allowing practical functionalities such as low shrinkage volume and stress [11], desired flowability (or viscosity) [12,13], shade [14,15], and good biocompatibility [16,17] to be obtained for various clinical applications. Several factors, including weight fraction, shape and size, orientation, and dispersion of the fillers in the resin matrix, determine the characteristics of the designed composite systems.

RBCs have been the most commonly used restorative materials in dentistry. Due to their good aesthetic and mechanical properties, they are used as direct or indirect restorations, pit and fissure sealants, cavity liners, veneers, crowns, endodontic sealers, and orthodontic devices [2].

Depending on the particle size, the composites are classified as macrofilled, microfilled, nanofilled, and hybrid (micro-hybrid and nano-hybrid). Nanofilled and nanohybrid RBCs have high polishing ability comparable to that of the enamel, good wear resistance, and transparency [6,7]. Due to their increased aesthetics, strength, and durability, they are increasingly preferred by clinicians as a universal restorative material for both anterior and posterior restorations [18].

Increasing the filler load in an RBC improves its overall physical properties as well as resistance to the functional wear placed on the restorative material. The material’s viscosity is directly affected, as the increase in filler loading will result in a sculptable higher-viscosity material, while less filler material will result in a flowable low-viscosity-based material. The main advantages of flowable composites include high wettability of the tooth surface, the ability to form layers with a minimum thickness, high flexibility, radiopacity, and different colors of the material.

The challenges flowable composites faced were in the areas of strength and fracture toughness, wear resistance, and polymerization shrinkage as well as in modulus of elasticity [18,19].

To maximize the physical, mechanical, and biological performance of the composites, strong interfacial reactions between fillers and resin matrix are essential. The coupling agents silane monomers, which contain organic–inorganic functional groups and can chemically bridge the inorganic fillers to organic resins to enhance the interfacial bonding, are most widely used to modify the surface of the filler materials [20,21,22,23]. Their role is to provide a strong and stable chemical bond between the organic matrix and the inorganic fillers [3,4,24].

Two types of initiators are mainly used in RBCs—benzoyl peroxide in self-cured composites and most often camphorquinone (QC) in light-cured composites, as dual-cured materials contain both of them. Because of QC’s intense yellow color, alternative lighter-colored initiators that completely bleach out after photopolymerization have been recently promoted. These include phenyl propane-dione (PPD), acyl phosphine oxide (APO), and Ivocerin [25].

Conventional RBCs are applied using the incremental technique (2 mm thick composite layer), which is a time-consuming process and may result in inaccuracies. To simplify the procedure, manufacturers created bulk-fill composites which enabled placing layers up to 4 mm while ensuring sufficient depth of cure. This is achieved by optimization of the photoinitiator system, modification of fillers (larger size or higher particle translucency), or inclusion of various chemicals in the composition [26,27]. Flowable bulk-fill composites have lower filler content, resulting in poorer mechanical properties, so they should not be used as a surface layer of the filling, which is exposed to direct chewing load [28]. The application of bulk-fill composites in posterior restorations reduces cusp deflection [29,30,31] and polymerization stress [3,32], thus increasing the fracture resistance of the restoration and hard dental tissues. Bulk-fill materials contain specially patented photoinitiators. One of them is based on germanium and is commercially named Ivocerin. This highly reactive photoinitiator, compared to standard photoinitiators such as CQ, works by shortening the curing time and allows the curing light to penetrate up to 4 mm. [33,34,35,36,37,38].

RBCs are in the plastic phase, and their hardening occurs due to the visible light-initiated cross-linking of resin monomers into a three-dimensional polymer network [39].

A high degree of composite polymerization is essential for optimal physical properties and biocompatibility [40]. The conversion of monomers to the polymer is never complete and reaches up to 75%. At the beginning of light irradiation, photoinitiators are activated and turn into free radicals. The collision of free-radical initiators activates the monomers, forms covalent bonds between carbon atoms, and forms long-chain polymers. The lengthening and the interaction of the polymer chains cause an increase in the viscosity and the rigidity of the composite paste. Within a rapidly stiffening structure, certain unreacted monomers remain trapped. Residual unconverted methacrylate groups which may reside in lower parts of poorly polymerized composite fillings present not only cytotoxic and genotoxic risks, but also their solubility might cause the formation of voids and the occurrence of secondary caries [39].

In the last decade, there has been significant development of light-curing units as well as the introduction of various light-curing protocols. Conventional light with radiant exitance of about 1000 mW/cm^2^ has been most commonly used in clinical practice. Recently, high-intensity curing units have been put into practice, which use light intensity of over 2000 mW/cm^2^. There are a large number of factors that affect the quality of polymerization (light intensity, curing light distance, exposure time) [41,42,43]. With the use of high-intensity light, along with shortening the exposure time, there was a concern about increasing the polymerization shrinkage stress [44,45].

The successful polymerization of RBCs, characterized by the monomer–polymer conversion ratio, can be evaluated by their hardness. There is a positive correlation between the conversion ratio and hardness of RBCs. It was found that 80% bottom-to-top hardness ratio corresponds to a 90% conversion ratio. On the other hand, the wear and fracture resistance as well as the durability of the restoration are defined by the composite hardness. Higher MH values correlate with higher biocompatibility of composite fillings [3,46].

A group of authors reports that there are significant differences in the degree of conversion of the deeper layers of the RBCs in those polymerized with different curing intensities [47,48], while other authors present that the exposure time has a greater influence on the MH and the conversion ratio [49,50].

The MH of the RBCs is also influenced by the size, volume, and weight of the filler particles. A positive correlation between filler content (wt% and vol%) and surface hardness in dental composites was shown. It was found that RBCs containing nanofillers show higher values of MH [51]. Flowable composites with lower filler content and higher volume of the organic matrix usually show lower MH values, as well as higher levels of polymerization shrinkage [52].

The shade of RBCs also has an effect on MH and conversion ratio. It was observed that opaque materials and materials with high filler load, which exhibit stronger light scattering, consequently had a lower degree of conversion and lower MH. Conversely, translucent shades exhibit a higher degree of conversion and higher MH [39].

Microhardness is defined as the resistance against penetration or permanent indentation of the surface, which is a criterion for resistance to plastic deformation, and is calculated by using the applied force divided by the surface area of the indentation. The Vickers test is one of the most commonly used tests for testing MH [53].

The aim of this study was to compare the effect of HV and LV curing protocols on MH values and the MH bottom–top ratio, as well as the influence of the filler content (wt%, vol%) on MH, for conventional and bulk-fill composites, including composites designed for high-intensity light curing.

## 2. Materials and Methods

### 2.1. Tested Materials and Light-Curing Protocols

Five different resin composites were used in the research: 3 conventional and 2 bulk-fill composites (Table 1). The two tested composites (PFL and PFW) were designed for high-intensity light curing.

Two types of curing protocols were tested; the high-voltage protocol (HV protocol) involved light curing for 3 s with a radiant exitance of 3440 mW/cm^2^, while the conventional or low-voltage protocol (LV protocol) involved light curing for 10 s with radiant exitance of 1340 mW/cm^2^. An LED curing unit (Bluephase PowerCure, Ivoclar Vivadent, Schaan, Liechtenstein, emission wavelength range: 390–500nm) was used in this research.

### 2.2. Specimen Preparation

A total of 40 cylindrical composite specimens were made (6 mm diameter, height of 2 mm for conventional and 4 mm for bulk-fill composites), 8 samples for each composite (*n* = 8). Specimens were made in metal molds, open on both sides, on the bottom side flattened with glass plates, and on the top side covered with Mylar foils to obtain the smooth surface needed for the proper measurement of the MH [23]. Composite specimens were irradiated only from the top side (that was marked), according to the described curing protocols, and were stored in a dry and dark place for 24 h to complete the post-cure reaction [24]. The samples were subsequently polished on the top and bottom with a four-step coarse to superfine grain disc system (20 s per step) (Sof-Lex, 3M ESPE, St. Paul, MN, USA) at a speed of 15,000 rpm. Final polishing was performed with the Sof-Lex diamond polishing system, which consists of pre-polishing and diamond-impregnated polishing spirals that achieve a highly polished surface. Surface residues were removed by washing and drying the samples.

### 2.3. Microhardness Measurement Protocol

MH was measured on the top and bottom surfaces of the specimen using a digital microhardness tester (QNESS 60 M EVO, ATM Qness GmbH, Mammelzen, Germany) equipped with a Vickers diamond indenter and a microscope with a magnification of 20×.

**Figure 1:**
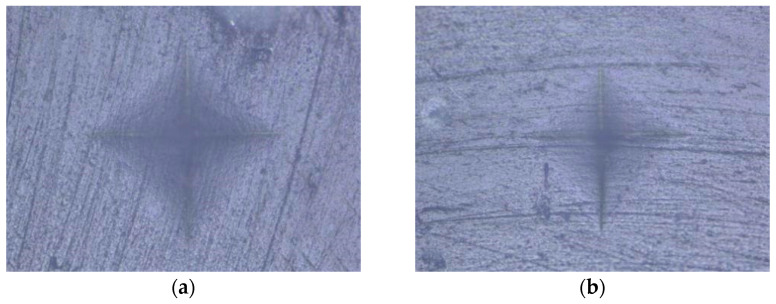
Evetric: (**a**) HV protocol; (**b**) LV protocol.

**Figure 2:**
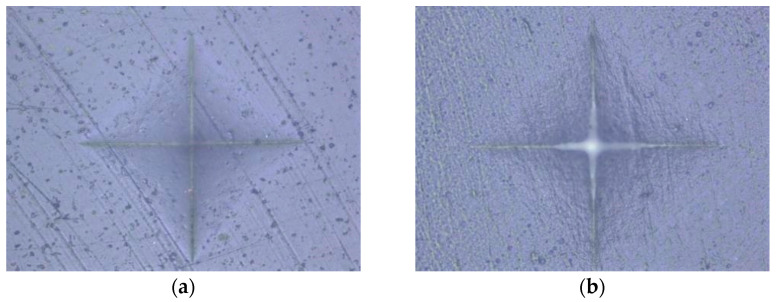
Tetric Evo Flow: (**a**) HV protocol; (**b**) LV protocol.

**Figure 3:**
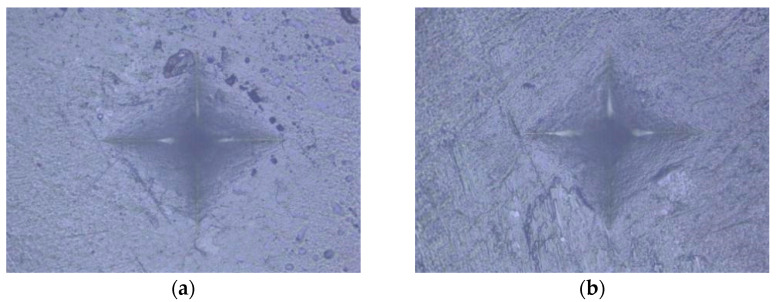
Tetric Power Fill: (**a**) HV protocol; (**b**) LV protocol.

**Figure 4:**
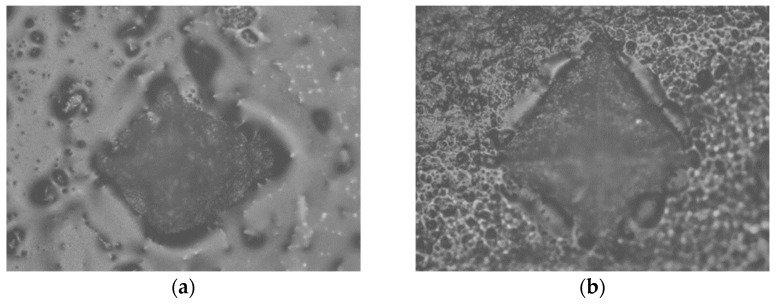
Tetric Power Flow: (**a**) HV protocol; (**b**) LV protocol.

**Figure 5:**
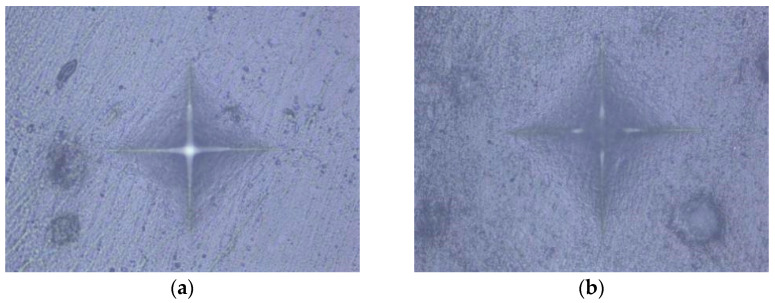
Tetric Prime: (**a**) HV protocol; (**b**) LV protocol.

An indentation in the shape of a diamond was made in the middle of the surface of the specimen under a load of 100 g for 20 s [55]. Based on the size of the impression, using the equation HV = 0.1891 × F/d^2^, where F is the load in N (newtons) and d is the mean value of the diagonals in mm (millimeters), the device using the integrated microscope automatically determined the MH value of the specimen surface (Figure 1, Figure 2, Figure 3, Figure 4 and Figure 5). The mean value of MH was obtained by measuring MH at 5 places on the surface of each sample (top and bottom).

### 2.4. Statistical Analysis

IBM SPSS v25 software and Microsoft Excel were used for the statistical analysis of the results, with a statistical significance level of α = 0.05. As a preliminary analysis, the Kolmogorov–Smirnov and Shapiro–Wilk tests were calculated to test the normality of the distribution. The obtained data were distributed normally; therefore, the use of parametric statistics was justified. The following analyses were performed: RMANOVA, Pearson’s correlation coefficient, and descriptive statistics. Repeated measures analysis of variance (RMANOVA) was used to examine the main effects of materials and curing protocols, as well as the interaction effect of materials and curing protocols on MH. Pearson’s correlation coefficient was used to test the relationship between MH and filler content (wt% and vol%).

## 3. Results

Table 2 shows the results of descriptive statistics: central tendency measure, standard deviation, and min and max functions for all tested variables.

In Table 3, the results show statistically significant effects of material and polymerization, as well as a significant interaction effect of the aforementioned variables. Statistical significance was present when MH was measured from both the upper and lower sides of the specimen. Partial eta-squared values represent a measure of the relative effect size. The results show that the effect of the variable polymerization is significantly lower compared to the second variable material, as well as compared to the interaction effect. This indicates that the differences in MH are mostly related to the variability in the material, rather than the variability in the curing light voltage (different curing protocols).

**Figure 6:**
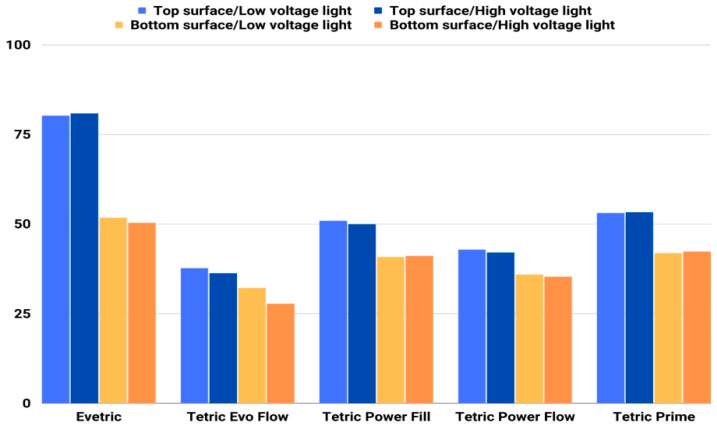
Initial MH of tested composites on top and bottom specimen surface (mean values).

Initial MH values measured on the top and bottom specimen surfaces are shown in Figure 6. The MH values ranged between 27.7 and 42.8 for the flowable and between 35.2 and 80.8 for the sculptable composites. The highest MH values were measured with the sculptable composite EVT from the top surface, while the lowest values were obtained with the flowable composite TEF. The biggest difference in the MH of top and bottom surfaces was observed with sculptable composite EVT, followed by TP and PFL. EVT with values of 80.0 and 80.8 on the top side and 51.7 and 50.3 on the bottom side of the specimen has a significantly higher MH value compared to other composites.

The lowest initial MH values, as well as the smallest difference in MH values of top and bottom surfaces, were measured for flowable composites. MH values of the top surface of the flowable composites (TEF and PFW) show similar values for both curing protocols. Higher initial MH was shown by PFW compared to TEF on both sides of the specimen.

Pearson’s correlation coefficient was used to test the relationship between MH and composite filler content (wt%, vol%). Correlations were made with MH values measured on the top surface of the specimen polymerized with two curing protocols [25]. A total of four correlations were tested, as shown in Table 3. It was determined that there is no statistically significant correlation, but it is important to emphasize that the obtained correlation coefficients are considered very high (Table 4).

**Figure 7:**
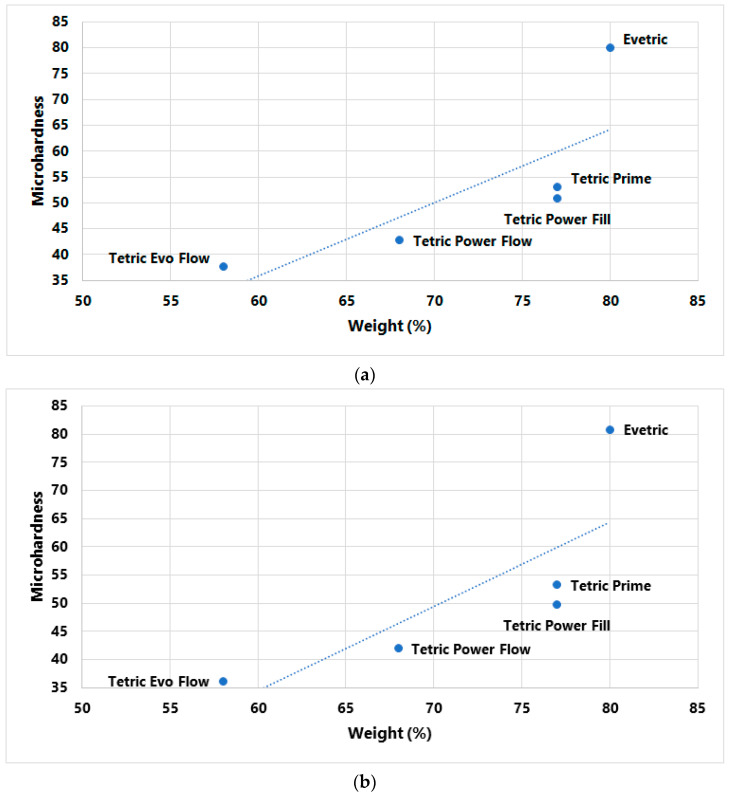
Plots of initial MH measured on top specimen surface vs. filler content (wt%) with (**a**) LV protocol and (**b**) HV protocol.

**Figure 8:**
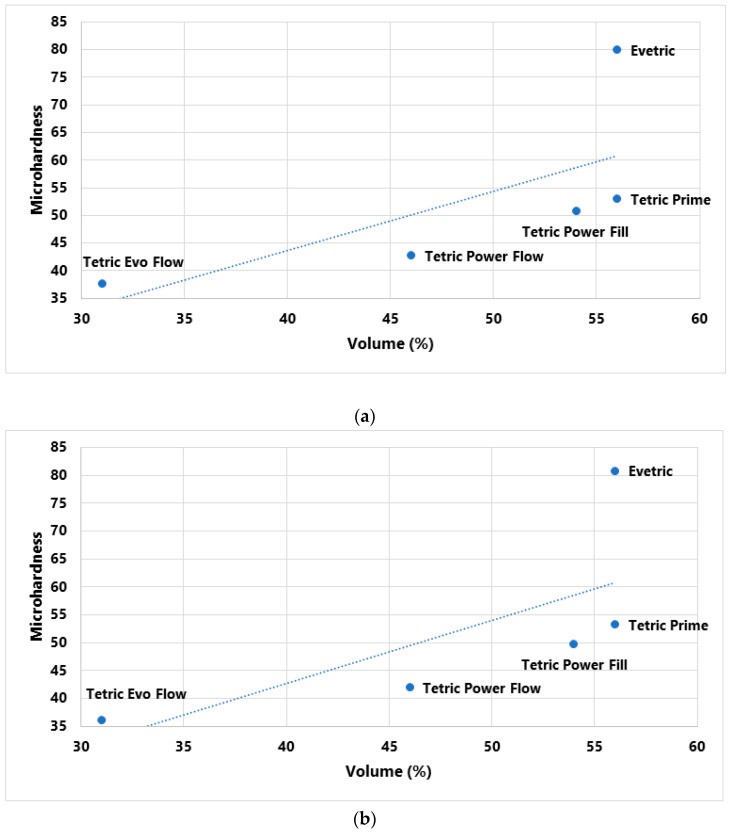
Plots of initial MH measured on top specimen surface vs. filler content (vol%) with (**a**) LV protocol and (**b**) HV protocol.

In Figure 7 and Figure 8, initial MH values measured on the top specimen surface are plotted as a function of filler content. No statistically positive correlation between MH values and filler wt% was established, but the correlation coefficient is very high, 0.78 for the LV protocol and 0.783 for the HV protocol. A comparatively weaker association was identified between MH and filler vol%; correlation coefficients were 0.694 for the LV protocol and 0.701 for the HV protocol.

**Figure 9:**
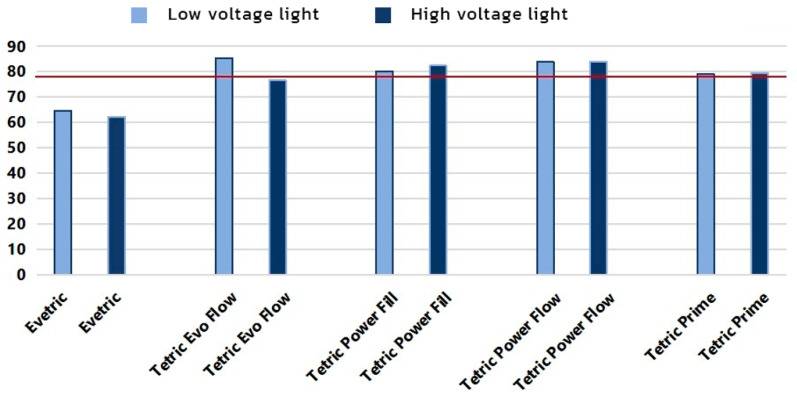
Bottom–top ratio (%) for initial MH. Red line marks 80% bottom/top MH threshold.

Figure 9 shows bottom/top ratios for initial MH values. The values of bottom/top ratios ranged between 61.5 and 84% for the HV protocol and between 65 and 86% for the LV protocol. EVT shows significantly reduced top/bottom ratio values of 61.5% for the HV protocol and 65% for the LV protocol, while the top/bottom ratios for TEF polymerized with the HV protocol and TP polymerized with the HV and LV protocols are close to the threshold of 80%. Bulk composites PFL and PFW show the best bottom/top MH ratio of all tested composites for both curing protocols.

## 4. Discussion

The improvement of the material properties of RBCs is essential to obtain reliable and long-lasting clinical results.

The surface properties of resin composites, roughness, and microhardness have gained great clinical importance, as they are related to the esthetics and function of restorations. The superficial microhardness of RBCs is important for the clinical success of restoration, since the higher the microhardness of restorative material, the better the resistance to surface wear and scratching. Inadequate polishing of RBCs results in periodontal disease and the development of secondary caries due to increased plaque accumulation, compromising long-term clinical success [36,56]. A Sof-Lex system was used in the present study to polish the surface of resin composites and prepare the sample for MH testing, as it has been reported that this allows obtaining a lower surface roughness compared with any other polishing system [36,57,58].

The successful polymerization of resin-based dental composites, expressed in their high hardness, depends on many factors related to the light-curing protocols, process parameters, and composition and properties of the restorative material. The hardness of the resin-based composites is defined by the monomer–polymer conversion ratio: the higher the polymerization ratio, the higher the hardness.

There is a large number of studies that examined the influence of curing protocols on the MH and top–bottom ratio of different composites. Most of them determined that light-curing protocols affect the micromechanical properties of different RBCs [59,60,61,62]. However, some studies did not confirm the influence of different curing protocols on the MH of RBCs [63,64]. Differences in the obtained results are probably due to the outcomes of such studies being highly dependent on the choice of material and testing procedures [55].

In this research, partial η^2^ values showed statistically significant effects of material and curing protocol, as well as a significant interaction effect of the aforementioned two variables on the initial MH on both specimen surfaces. The effect of the curing protocol variable is far lower than that of the material variable or the interaction effect, which means that changes in MH are more related to the variability in the material than to the variability in the curing protocol. The effect size of the curing protocol was higher on the bottom specimen surface compared to the top surface. Results presented by other authors also show that the material factor was more influential compared to curing protocols [65].

Compared to sculptable composites, flowable composites showed lower initial MH values with both curing protocols. The lowest MH values were measured for TEF. Par et al. [55] present differences in the MH values of flowable composites when using different curing protocols, while in this research, only TEF shows a modest reduction in MH resulting from the HV protocol. This can be explained by the reported finding that polymerization effectiveness tends to be more diminished by high-intensity light curing in flowable than in sculptable composites [66], which is why it is recommended to use high-intensity curing light with caution for flowable composites. Flowable composites, due to their low initial MH and filler content, have lower strength and durability, and the authors recommend the use of a sculptable composite over a flowable composite as a cover layer, especially in the region of strong masticatory forces [67,68].

The highest MH values were measured with EVT when using both protocols and were 80.0 and 80.8 for the top surface and 51.74 and 50.3 for the bottom surface, which are significantly higher compared to those of other composites. PFL and TP show similar MH values with both used protocols. Two of the composites investigated in this study (PFW and PFL) were specifically designed for use with the HV curing protocol. The investigated MH properties for these composites were mostly within the ranges obtained for other investigated composites of the corresponding viscosity (sculptable for PFL and flowable for PFW).

The goal of the research was also to determine how filler content (wt%, vol%) affects MH values. Filler content is considered to be the basic determinant related to the mechanical properties of the material. It is claimed that materials with high filler content would have higher surface hardness since, immediately after curing, the surface layer, mainly composed of the organic matrix, can further polymerize during polishing, thus increasing its strength [36]. The results of Pearson’s analysis in this research showed that there is no statistically significant correlation between the measured MH and filler content, but it is important to emphasize that the obtained correlation coefficients are considered very high for wt% (R = 0.78 and 0.783) and for vol% (R = 0.694 and 0.701) for the LV and HV protocols. The value of the correlation coefficient for filler weight percentage (wt%) is on the border of statistical significance. On scatterplots of initial MH vs. filler content, the highest deviation from the correlation line is shown for the conventional composite EVT, with both curing protocols. EVT has the highest percentage of filler content of all tested composites as well as the highest measured MH values. It is known that a higher percentage of inorganic filler reduces the transmission of visible light into deep layers, thereby reducing the polymerization in deeper layers [55], which can partially explain the large difference in the obtained MH value of the top and bottom specimen surfaces with EVT in this research.

The hardness on the bottom surface of the composites is lower compared to the top due to the lower polymerization ratio owing to the lower light energy input. According to the accepted criterion of bottom–top MH ratios, above 80% indicates acceptable polymerization throughout the composite layer [3,55,65].

In this research, suboptimal curing effectiveness was identified for EVT with a measured top/bottom ratio of 65% and 62.5% with LV and HV protocols, as well as for TEF (77% with HV protocol). Other investigated composites showed sufficient curing effectiveness. It was published that the decrease in MH in the deeper layers of specimens is significantly less with bulk-fill composites [43], which was also confirmed by this research. High matrix content and the presence of nanoparticles in the filler define a very high translucency of the unpolymerized bulk-fill composite, which allows the light to penetrate easily to the deepest layers of the restoration [3]. The best top–bottom ratio in this research was shown by the two bulk-fill composites also designed for the HV protocol, namely PFW (84% for LV and 83.5% for HV protocol) and PFL (80% for LV protocol and 81.5 % for HV protocol).

With the evolution of restorative materials, bulk-fill resin composites have emerged, offering improved physical and mechanical properties that depend on their composition, which varies according to manufacturers, as they can modify the organic matrix, size, and morphology of the filler particles to achieve adequate behavior.

A limitation of the present in vitro study is the fact that in a clinical situation, changes in the presence of saliva, enzymes, and changes in pH could affect MH over time.

## 5. Conclusions

The MH of five dental resin-based composites was investigated. The MH was evaluated with two curing protocols, with variations in the light intensity, curing time, and composite thickness. The following was concluded:

The MH of RBCs is more dependent on material composition than on light-curing protocol. The highest initial MH values were measured for sculptable conventional composites, and the lowest initial MH values were measured for flowable composites. Filler weight percentage has a greater influence on MH values compared to filler volume percentage. The bottom/top ratio showed values over 80% for bulk composites (both sculptable and flowable), while for conventional composites, borderline or suboptimal values were measured for both curing protocols. The conventional flowable composite showed a reduction in MH resulting from the HV protocol. The tested bulk-fill composites can be safely used up to at least 4 mm incremental thickness.

## Figures and Tables

**Table 1:** Resin composites investigated in the study [54].

Composite Viscosity	Composite Type	Composite Name	Resin Matrix	Filler Content (wt%/vol%)	Manufacturer
Sculptable	Conventional	Evetric (EVT)	UDMA, Bis-GMA, Bis-EMA	80/56	Ivoclar Vivadent, Schaan, Liechtenstein
		Tetric Prime (TP)	Bis-GMA, UDMA, Bis-EMA, D3MA	77/56	
	Bulk-fill	Tetric Power Fill (PFL)	Bis-GMA, Bis-EMA, UDMA, propoxylated bisphenol A dimethacrylate, DCP, β-allyl sulfone AFCT agent	77/54	
Flowable	Conventional	Tetric Evo Flow (TEF)	Bis-GMA, UDMA, decandioldimethacrylate	58/31	
	Bulk-fill	Tetric Power Flow (PFW)	Bis-GMA, Bis-EMA, UDMA	68/46	

**Table 2:** Descriptive statistics data.

Variable	Average	Standard Deviation	Min	Max
Evetric	65.73	15.15	49.10	81.70
Tetric Evo Flow	33.42	4.01	26.80	38.70
Tetric Power Fill	45.64	4.88	40.20	51.60
Tetric Power Flow	38.99	3.57	34.60	43.80
Tetric Prime	47.60	5.80	40.90	55.00
3 s curing	46.67	13.12	31.10	81.30
Conventional curing	45.88	13.94	26.80	81.70
Top surface	52.65	15.21	35.60	81.70
Bottom surface	39.90	7.25	26.80	53.60

**Table 3:** RMANOVA results (*p*-values and partial η^2^ values).

	Top Surface		Bottom Surface	
	*p*	Partial η^2^	*p*	Partial η^2^
Material	<0.000	0.998	<0.000	0.991
Curing protocol	<0.039	0.195	<0.000	0.515
Material × curing protocol	<0.011	0.463	<0.000	0.714

**Table 4:** Pearson’s correlation coefficients.

Filler Content and LV/HV Curing Protocol	*p*-Value	Correlation Coefficient (R)
wt% LV protocol	0.120	0.780
wt% HV protocol	0.117	0.783
vol% LV protocol	0.194	0.694
vol% HV protocol	0.188	0.701

## Data Availability

The data presented in this study are available on request from the corresponding author.
